# Should there be a standardised approach to the diagnostic workup of suspected adult encephalitis? a case series from Australia

**DOI:** 10.1186/1471-2334-10-353

**Published:** 2010-12-15

**Authors:** Clare Huppatz, Yash Gawarikar, Chris Levi, Paul M Kelly, David Williams, Craig Dalton, Peter Massey, Rodney Givney, David N Durrheim

**Affiliations:** 1 Hunter New England Population Health, NSW Health, Newcastle, New South Wales, Australia; 2 National Centre for Epidemiology and Population Health, Australian National University, Canberra, Australia; 3 John Hunter Hospital, NSW Health, Newcastle, New South Wales, Australia; 4 University of Newcastle, New South Wales, Australia; 5 Hunter Medical Research Institute, Newcastle, New South Wales, Australia; 6 Hunter Area Pathology Service, Pathology North, John Hunter Hospital, NSW Health, New South Wales, Australia

## Abstract

**Background:**

The clinical diagnosis of encephalitis is often difficult and identification of a causative organism is infrequent. The encephalitis syndrome may herald the emergence of novel pathogens with outbreak potential. Individual treatment and an effective public health response rely on identifying a specific pathogen. In Australia there have been no studies to try to improve the identification rate of encephalitis pathogens. This study aims to review the diagnostic assessment of adult suspected encephalitis cases.

**Methods:**

A retrospective clinical audit was performed, of all adult encephalitis presentations between July 1998 and December 2007 to the three hospitals with adult neurological services in the Hunter New England area, northern New South Wales, Australia. Case notes were examined for evidence of relevant history taking, clinical features, physical examination, laboratory and neuroradiology investigations, and outcomes.

**Results:**

A total of 74 cases were included in the case series. Amongst suspected encephalitis cases, presenting symptoms and signs included fever (77.0%), headache (62.1%), altered consciousness (63.5%), lethargy (32.4%), seizures (25.7%), focal neurological deficits (31.1%) and photophobia (17.6%). The most common diagnostic laboratory test performed was cerebrospinal fluid (CSF) analysis (n = 67, 91%). Herpes virus polymerase chain reaction (n = 53, 71.6%) and cryptococcal antigen (n = 46, 62.2%) were the antigenic tests most regularly performed on CSF. Neuroradiological procedures employed were computerized tomographic brain scanning (n = 68, 91.9%) and magnetic resonance imaging of the brain (n = 35, 47.3%). Thirty-five patients (47.3%) had electroencephalograms. The treating clinicians suspected a specific causative organism in 14/74 cases (18.9%), of which nine (12.1%) were confirmed by laboratory testing.

**Conclusions:**

The diagnostic assessment of patients with suspected encephalitis was not standardised. Appropriate assessment is necessary to exclude treatable agents and identify pathogens warranting public health interventions, such as those transmitted by mosquitoes and those that are vaccine preventable. An algorithm and guidelines for the diagnostic workup of encephalitis cases would assist in optimising laboratory testing so that clinical management can be best tailored to the pathogen, and appropriate public health measures implemented.

## Background

Encephalitis is an uncommon but important clinical and public health syndrome. Clinically, the diagnosis is frequently difficult, treatments are limited and outcomes often include serious morbidity or death[1]. From a public health viewpoint, encephalitis is a syndrome that can herald the emergence of novel pathogens with outbreak potential[2] and it is not currently under routine surveillance in Australia. Of significance to both clinicians and public health specialists is that pathogen identification in the majority of cases of encephalitis remains elusive[3,4].

Encephalitis is defined as inflammation of the brain parenchyma and presents with clinical evidence of brain dysfunction[5-7]. The classic clinical encephalitis triad is "headache, fever and altered consciousness" with a range of other possible clinical symptoms, including focal neurological signs, seizures and altered behaviour[8,9]. Diagnosis is based on a combination of clinical features, laboratory tests and neuroradiological imaging[6,7,10].

Infectious encephalitis can be caused by numerous pathogens that vary by geographical location. Viruses are the most common causative agents with herpes simplex virus (HSV) the most regularly implicated in developed countries[11,12]. A complete list of potential pathogens is difficult to compile, but has been attempted [8,13,14]. Diagnosis of the causative agent is important for treatment, prognosis and public health interventions. Effective treatment is available for some bacterial and parasitic pathogens, as well as herpes simplex virus, where treatment with acyclovir reduces mortality from 70% to 20-30%[15,16]. For many other pathogens treatment is supportive, however, prognosis differs by causative agent[6]. The public health response depends on the geographic location but in Australia early detection of certain causative pathogens, such as Murray Valley Encephalitis (MVE), Australian Bat Lyssavirus (ABL), Kunjin virus, and Hendra virus would lead to measures aimed at limiting further transmission in the community[2].

Pathogen identification rates for patients hospitalised with encephalitis vary between countries, but have been reported in 40% of hospitalised patients in the United States (US)[17] and 20-40% of encephalitis related deaths in the US[18] and United Kingdom (UK)[19]. In NSW, a review of 18 years of hospital data found that only 30% of encephalitis patients were discharged with a pathogen diagnosis[4]. Forty-eight percent of encephalitis related deaths in Australia have a pathogen diagnosis[3].

In Australia there have been no studies to try to improve the identification rate of encephalitis pathogens and there is currently no widely accepted diagnostic algorithm to guide clinicians in their workup. The Infectious Diseases Society of America (IDSA) published guidelines for the management of encephalitis in 2008 which included a standardised diagnostic evaluation, but the relevance in Australia needs to be determined[20]. The Health Protection Agency (HPA) guidelines for the investigation of viral encephalitis in the UK are currently under review[21] and the HPA is leading a multi-centre prospective study to assess the aetiology of encephalitis in England. A recent study from France suggested that a standardised approach to pathogen diagnosis may improve the pathogen identification rate[22].

This study aims to review the diagnostic work-up currently performed by clinicians investigating suspected adult encephalitis cases in Australia.

## Methods

Adult encephalitis cases were identified at the John Hunter, Maitland and Tamworth Regional Referral Hospitals, which provide all referral neurological services for the Hunter and New England areas of northern New South Wales (NSW) (population 814,146), from July 1998-December 2007 using the NSW Health hospital statistics database. The database was searched using all ICD-10 codes pertaining to encephalitis. The search selected only patients ≥ 18 years who were admitted to hospital and given a primary discharge code for encephalitis. Cases were excluded if the treating physician documented that the case did not have encephalitis at the time of discharge (coding error), if the patient was transferred to another facility (other than one involved in the study) prior to a diagnostic work-up being performed, if the case had transverse myelitis (a shared encephalitis code), or if an encephalitis diagnosis was already made prior to admission, negating the need for a diagnostic work-up.

The patient records of suspected encephalitis cases were individually reviewed by one of two members of the research team (both clinicians), using a specially designed audit tool (CH, YG). Information was extracted regarding: length of hospitalisation, Intensive Care Unit (ICU) stay and ventilatory support (as appropriate), signs and symptoms at the time of admission, relevant exposure history, laboratory and neuroimaging results, the clinical diagnosis and outcome (using the Glasgow Outcome Scale) at the end of the episode of care. If a patient chart was difficult to read or interpret, consensus regarding the finding was reached between these two researchers. Laboratory and neuroimaging results were sought in hospital's computerised databases as well as in the patient record.

### Signs and symptoms at time of admission

The following clinical signs and symptoms were recorded for each case: headache, fever, altered consciousness (including irritability and/or coma), lethargy, seizures, abnormal behaviour, focal neurological signs, neck stiffness, rash, photophobia, myalgia and/or arthralgia. Signs and symptoms were recorded in the patient record as present "at presentation" if there was a record of their presence within the first 48 hours of admission or the patient/next of kin reported them in the 24 hours prior to admission.

### History of relevant exposures/risk factors

Each patient record was searched for a documented history of the following: occupation, overseas travel, domestic travel, contact with animals, exposure to insects, contact with bats, water sports/water exposure, and other exposures considered relevant to the treating clinicians.

### Laboratory results

All blood tests, lumbar puncture, brain biopsy and autopsy results were recorded as performed or not performed, and whether the outcome led to a pathogen diagnosis. Additionally, the CSF result was recorded as consistent with viral encephalitis if the result demonstrated raised WCC, with > 50% monocytes, raised protein and normal glucose[23].

All testing performed to attempt to identify a specific pathogen was recorded. Tests for specific pathogens performed were compared to a predetermined list of 27 "possible encephalitis pathogens". The list of "possible encephalitis pathogens" of relevance in an Australian setting was compiled by the authors, who formed a Reference Panel, chosen for their expertise in neurology, microbiology, and/or public health.

### Neuroimaging results

For each case of encephalitis, a record of all investigations by computerized tomography (CT) and magnetic resonance imaging (MRI) was recorded. It was further documented whether the result was "consistent with encephalitis" based on the radiology report.

### Electroencephalogram (EEG)

For each case, EEGs performed were recorded and whether the result was consistent with encephalitis.

### Clinical diagnosis

Each patient record was examined to determine the final diagnosis made by the treating clinician. Where possible, the discharge summary was used for this purpose. Where a discharge summary was not available, the final notes recorded by treating clinicians were reviewed to determine the final diagnosis. Where the clinician had ascribed a particular pathogen as the cause of the patient's encephalitis, this was noted and compared to the relevant laboratory findings.

### Outcome (using the Glasgow Outcome Scale)

Outcome for each case of suspected encephalitis was recorded using the Glasgow Outcome Scale, as either normal (GOS category 5), disabled (GOS categories 2, 3, 4) or deceased (GOS category 1).

### Ethics

Ethical approval was given by the Hunter New England and the Australian National University Human Research Ethics Committees.

## Results

A total of 104 adult patients with a discharge diagnosis of encephalitis were admitted to the John Hunter Hospital, Maitland Hospital and the Tamworth Regional Referral Hospital between July 1998 and December 2007. Their encephalitis ICD-10 codes are shown in Table 1. Thirty patients were excluded from our study due to: coding errors (12 cases), transfer to another facility prior to diagnostic work-up (2 cases), transverse myelitis (13 cases), and encephalitis diagnosis made prior to admission (3 cases). Of the 74 cases included, 42 were from the John Hunter Hospital, 18 from the Maitland Hospital and 14 from the Tamworth Regional Referral Hospital.

**Table 1:** ICD-10 code diagnosis and the number of cases with a laboratory confirmed pathogen diagnosis

ICD-10 Code	Code name	Cases	Pathogen confirmed by laboratory test
**Codes for "known" pathogens**			
A321	Listerial meningitis & meningoencephalitis	3	3

A858	Other specified viral encephalitis	3	2

B004	Herpesviral encephalitis	10	2

B011	Varicella encephalitis	1	1

B020	Zoster encephalitis	3	1

G052	Encephalitis, myelitis & encephalomyelitis-other infectious and parasitic diseases	1	0

G040	Acute disseminated encephalitis	1	N/A

**Subtotal for "known" pathogens**		**22**	**9**

**Codes for "unknown" pathogens**			

G048	Other encephalitis, myelitis and encephalomyelitis	1	N/A

G049	Encephalitis, myelitis and encephalomyelitis, unspecified	10	N/A

A86	Unspecified viral encephalitis	41	N/A

**Subtotal ("unknown" pathogens)**		**52**	**N/A**

**Grand Total**		**74**	**9**

The median age of cases was 49.2 years (IQR 35.5-65.5) and 41 cases (55%) were male. There were no apparent geographical clusters of cases by postcode, nor was there a difference in admission rates by season (or month). The mean length of stay was 12 days (range 1-63 days), with 26/74 (35%) patients requiring an ICU admission and 17/74 (23%) ventilatory support.

### History of relevant exposures/risk factors

An occupational history was documented in 44/74 (57.5%) cases, a history of domestic travel in 16/74 (20.0%) cases, overseas travel in 10/74 (12.5%) cases, exposure to animals in 9/74 (11.3%) cases and exposure to water sports in 1/74 cases (1.5%). Exposure to insects and/or bats was not recorded for any patient and other relevant encephalitis risk factor histories were either not elicited or documented very infrequently in patient notes.

### Signs and symptoms at time of admission

The classical encephalitis triad of headache, fever and altered conscious state (including coma and/or irritability) was present in 26/74 (35.1%) cases (Table 2).

**Table 2:** Signs and symptoms "at presentation"* for all hospitalised adult encephalitis cases in three Hunter New England hospitals, Australia, July 1998-December 2007

Symptoms at presentation	Cases, n = 74 (%)
**Fever**	57 (77.0%)

**Altered Consciousness State (ACS) including irritability and/or coma**	51 (68.9%)

**Headache**	46 (62.1%)

**Encephalitis "triad"** **(headache, fever, ACS)**	26 (35.1%)

**Other symptoms:**	

**Lethargy**	24 (32.4%)

**Focal neurological signs**	23 (31.1%)

**Seizures**	19 (25.7%)

**Photophobia**	13 (17.6%)

**Neck stiffness**	11 (14.9%)

**Abnormal behaviour**	9 (12.1%)

**Rash**	7 (9.5%)

**Myalgia and/or arthralgia**	2 (2.7%)

*a sign/symptom was considered to be present "at presentation" if the patient/next of kin reported to have had the sign/symptom in the 24 hours prior to presentation or if it was documented in the patient record during the first 48 hours of their admission.

### Neuroimaging

A CT brain was performed in 68/74 cases (91.9%) and an MRI brain in 35/74 cases (47.3%). Two of the CT scans and eight of the MRI scans were reported as "consistent with encephalitis".

### Electroencephalogram

An EEG was done in 36/74 cases (48.6%), of which only four showed focal temporal lobe activity consistent with Herpes simplex encephalitis. Two of these patients had corresponding MRI abnormalities.

### Laboratory tests

A CSF sample was taken from 67/74 patients (90.5%) with encephalitis. Twenty-nine of these 67 cases (43.3% of the total) had both a high monocyte count and raised protein, consistent with viral encephalitis. Table 1 shows the ICD10 codes for the cases (n = 9) for which a laboratory test confirmed the discharge diagnosis.

Table 3 records the specific encephalitis pathogens for which tests were ordered on CSF or blood, as appropriate, and their relative frequency. The average number of tests ordered for the study patients was three per patient.

**Table 3:** Specific encephalitis pathogens and frequency of testing

Viral pathogens	Testing frequency, n = 74 (%)	Bacterial pathogens	Testing frequency, n = 74 (%)
Adenovirus	5 (6.8%)	Bartonella sp	1 (1.4%)

Australian Bat Lyssavirus	0	Chlamydia species	5 (6.8%)

Cytomegalovirus	18 (24.3%)	Legionella pneumonia	4 (5.4%)

Enterovirus group	20 (27.0%)	Mycoplasma pneumoniae	9 (12.2%)

Epstein-Barr virus	16 (21.6%)	Q fever	10 (13.5%)

Flavivirus	11 (14.9%)		

Herpes simplex virus	53 (71.6%)	**Other pathogens**	**Testing frequency, n = 74 (%)**

Influenza A & B viruses	6 (8.1%)	Cryptococcus	46 (62.2%)

Japanese Encephalitis	0	Mycobactrium tuberculosis	11 (14.9%)

Kunjin	2 (2.7%)	Rickettsiae	2 (2.7%)

Measles virus	1 (1.4%)	Toxoplasma	7 (9.5%)

Mumps virus	0		

Murray Valley Encephalitis	5 (6.8%)		

Rubella	2 (2.7%)		

Varicella zoster virus	14 (18.9%)		

### Outcome (using the Glasgow Outcome Scale, GOS)

Four patients in our study died during their hospital stay, 25/74 (33.8%) cases were "disabled" (GOS 2, 3 or 4) at the time of their discharge and 40/74 (54.1%) were classified as "normal" (GOS 5). There were five cases for which a decision regarding their GOS outcome could not be made due to missing information in their patient record.

## Discussion

This hospital audit found that the investigation of suspected adult encephalitis cases in northern NSW, Australia by a specialised neurological service was not standardised. Relevant patient history that would assist diagnostic investigation was seldom recorded in the patient record and only a limited number of pathogens were commonly sought.

Documented history taking in medical records reflected inadequate exploration of many potential encephalitis risk factors. Although this may have been asked, there was no formal record excluding important risk exposures. This may represent missed opportunities for assisting with pathogen diagnosis and/or public health action.

These audit results found that the majority of the 27 pathogens, which the multidisciplinary Reference Group considered important to exclude in patients with suspected encephalitis in Australia, were tested for infrequently, with each patient having an average of only three specific pathogen tests done. The most commonly tested pathogens were HSV and Cryptococcus, presumably because these pathogens are easily tested and amenable to treatment. Among the nine patients with identified pathogens, herpesviruses were the leading viral cause identified in our study (two cases due to HSV and one case of varicella zoster virus), possibly reflecting the frequency of testing. Three cases were found to be due to Listeria, with no other bacterial cause for encephalitis found. Whether an expanded standardised testing regime would identify more causative organisms warrants further investigation.

Fever, headache and altered consciousness were the most common presenting symptoms in patients with encephalitis but the clinical triad[8,9] of all three features was only seen in 26/74 (35.1%) patients. Seizures (25.7%) and focal neurological signs (35.1%) were the other relatively frequent presenting clinical features. Thus nearly two thirds of these patients with possible encephalitis presented with a variable constellation of symptoms making this syndrome a diagnostic challenge. Other conditions, particularly autoimmune encephalitis may present with a clinical picture similar to viral encephalitis[24].

Although there were only four deaths in the study population, 25 patients (33.8%) had significant disability at discharge from hospital with a Glasgow Outcome Scale of 2, 3 or 4. Incidence and severity of neurological sequelae vary depending on the aetiological agent causing encephalitis[6]. As there was a low rate of pathogen identification, this study is unable to speculate about clinical severity of different pathogens.

In general, studies of coded hospital discharge data have shown a poor rate of encephalitis pathogen diagnosis[4,17,19]. Our hospital audit found that although 22/74 (30%) cases had a "known" pathogen ICD-10 code, the number of laboratory confirmed pathogen diagnoses was fewer; 22 cases had an ICD-10 code for a "known pathogen", however, only 9/22 (41% of patients with "known" pathogen codes) actually had a laboratory confirmed specific pathogen diagnosis. Thus more than 85% of cases had no specific aetiology confirmed. This finding casts doubt on previous studies of hospital coded data, suggesting that the true rate of pathogen diagnosis could be much lower than that reported. This highlights the need to explore the use of a suitable clinical algorithm for the diagnosis of encephalitis cases. The guideline would need to be regionally verified, given the unique pathogens found in Australia. It has been suggested that such regional guidelines would enhance pathogen diagnosis rates and may lead to the discovery of novel infectious and non-infectious entities[25].

There are several weaknesses inherent in a retrospective study of this nature. Although we report on the medical history of each encephalitis patient and the symptomatology at time of presentation, our findings are limited to information from the patients' records. Certainly some aspects of history may have been taken and some symptoms or signs may have been present, but not recorded in the patient record. Since the study covers a 10-year period the increased availability of molecular diagnostic methods may have resulted in a systematic change in diagnostic approaches. In addition there would be value in confirming these findings in a larger multi-centre study.

## Conclusions

Encephalitis is a serious disease with consequences not only to the individual, but specific encephalitis-causing pathogens have significant public health implications. From a clinical perspective, it is important to exclude treatable agents, such as *Herpes virus *and *Cryptococcus *in all patients. It would also be useful to exclude other common causes of encephalitis. From a public health perspective, some pathogens have particular significance to the wider community and warrant public health interventions, such as those transmitted by mosquitoes (MVE, kunjin, flavivirus) and bats (ABL), and those that are vaccine preventable (influenza, mumps, measles, rubella). Australia may benefit from guidelines to aid clinicians in making a diagnosis of specific pathogens causing encephalitis. An important next line of research would be to formulate and trial an algorithm for diagnostic work-up. If such an algorithm still delivers low pathogen diagnostic yield, it would be important to consider novel testing techniques to search for new pathogens.

## Competing interests

The authors declare that they have no competing interests.

## Authors' contributions

CH participated in study design; collected, analyzed and interpreted data; and drafted manuscript. YG collected, assisted with analysis and interpretation of data; and drafted manuscript. CL participated in study design; reviewed data analysis and interpretation; and revised manuscript for important intellectual content. PMK participated in study design; participated in data interpretation; and revised manuscript for important intellectual content. DW participated in study design; reviewed data analysis and interpretation; and revised manuscript for important intellectual content. CD participated in study design; reviewed data interpretation; and revised manuscript for important intellectual content. PM participated in study design; reviewed data analysis and interpretation; and revised manuscript for important intellectual content. RG participated in study design; reviewed data analysis and interpretation; and revised manuscript for important intellectual content. DND conceived study and participated in the study design; supervised collection of data and analysis; participated in data interpretation and writing of manuscript.

All authors have read and approved the final version of the manuscript.

## Pre-publication history

The pre-publication history for this paper can be accessed here:

http://www.biomedcentral.com/1471-2334/10/353/prepub
